# Supplementary material for: Manipulation on active electronic states of metastable phase β-NiMoO4 for large current density hydrogen evolution
Source: Nat Commun. 2021 Oct 13;12:5960. doi: 10.1038/s41467-021-26256-1 (PMC8514534; doi:10.1038/s41467-021-26256-1)
Supplement: Supplementary file 1 — Supplementary Information [file 41467_2021_26256_MOESM1_ESM.pdf]

SUPPLEMENTARY INFORMATION

Manipulation on Active Electronic States of Metastable Phase β-$NiMoO_4$ for Large Current Density Hydrogen Evolution

Zengyao Wang[#1,2], Jiyi Chen[#1,3,4], Erhong Song[#5], Ning Wang[6] Juncai Dong[7], Xiang Zhang[8], Pulickel M. Ajayan[8], Wei Yao[1], Chenfeng Wang[1], Jianjun Liu[5], Jianfeng Shen*[1] and Mingxin Ye*[1]*

[1]Institute of Special Materials and Technology, Fudan University, Shanghai, China
[2]Department of Chemistry, Fudan University, Shanghai, China
[3]Joint School of National University of Singapore and Tianjin University, International Campus of Tianjin University, Binhai New City, Fuzhou, China
[4]Department of Chemical and Biomolecular Engineering, National University of Singapore, Singapore
[5]State Key Laboratory of High Performance Ceramics and Superfine Microstructure, Shanghai Institute of Ceramics, Chinese Academy of Sciences, Shanghai, China
[6]Institute of Environment and Life, Beijing University of Technology, Beijing, PR China
[7]Beijing Synchrotron Radiation Facility, Institute of High Energy Physics, Chinese Academy of Science, Beijing, China
[8]Department of Materials Science and Nano Engineering, Rice University, Houston, USA

[#]These authors contribute equally to this paper.
[*]Correspondence to: mxye@fudan.edu.cn, jfshen@fudan.edu.cn, jliu@mail.sic.ac.cn.

Table of Content:

1. Supplementary Methods

2. Supplementary Figures 1-38.

3. Supplementary Tables 1-6.

Supplementary Methods

Turnover frequency (TOF) calculation. TOF value was calculated by the previously reported method[1,2] and the detailed process was shown as follows:

(1) To receive TOF, the following formula was used:

$$\mathrm{TOF} = \frac{\text{the number of total hydrogen turnovers per geometric area}}{\text{the number of active sites per geometric area}} \quad (1)$$

The total number of hydrogen turnovers was calculated from the current density:

$$\mathrm{No.\ (H_2)} = \left(j\,\frac{\mathrm{mA}}{\mathrm{cm^2_{geo}}}\right)\left(\frac{1\ \mathrm{C\ s^{-1}}}{1000\ \mathrm{mA}}\right)\left(\frac{1\ \mathrm{mol\ e^{-1}}}{96485.3\ \mathrm{C}}\right)\left(\frac{1\ \mathrm{mol\ H_2}}{2\ \mathrm{mol\ e^-}}\right)\left(\frac{6.022\times 10^{23}\ \mathrm{H_2\ molecules}}{1\ \mathrm{mol\ H_2}}\right)$$

$$= 3.12\times10^{15}\,\frac{\mathrm{H_2/s}}{\mathrm{cm^2_{geo}}}\ \text{per}\ \frac{\mathrm{mA}}{\mathrm{cm^2_{geo}}} \quad (2)$$

(2) The number of the real active sites per real surface area is demonstrated by the following calculation formula from the unit cell of the β-$NiMoO_4$ crystal structure (the number of O atoms is known by the DFT calculation).

$$\text{No. of active sites} = \left(\frac{\text{No. of atoms/(unit cell)}}{\text{Volume/(unit cell)}}\right)^{\frac{2}{3}} \quad (3)$$

The number of active sites per real surface area for the β-$NiMoO_4$ are calculated as the following:

$$\text{No. of active sites } (\beta\text{-NiMoO}_4) = \left(\frac{4\,\frac{\text{atoms}}{\text{unit cell}}}{631.4\,\frac{\text{Å}^3}{\text{unit cell}}}\right)^{\frac{2}{3}} = 1.36\times10^{14}\text{atoms cm}^{-2}_{\text{real}} \quad (4)$$

(3) The real surface area for HER is demonstrated by the electrochemical active surface area (ECSA), which can be converted from the specific capacitance. According to these reported references, the specific capacitance for a flat surface is generally discovered among 20 to 60 µF cm^{2}_{geo}, and we used the average value of 40 µF cm^{2}_{geo} for the TOF calculation for β-$NiMoO_4$.

$$A_{\mathrm{ECSA}} = \frac{\text{specific capacitance}}{40\ \mu\mathrm{F\ cm^{-2}_{geo}}\ \text{per}\ \mathrm{cm^{2}_{ECSA}}} \quad (5)$$

$$A_{\mathrm{ECSA}}(\text{P-NiMoHZ}) = \frac{103.8\ \mathrm{mF\ cm^{-2}_{geo}}}{40\ \mu\mathrm{F\ cm^{-2}_{geo}}\ \text{per}\ \mathrm{cm^{2}_{ECSA}}} = 2595\ \mathrm{cm^{2}_{ECSA}} \quad (6)$$

(4) Finally, the plot of current density can be converted into a TOF plot through the following formula.

$$\mathrm{TOF} = \frac{(3.12\times10^{15}\ \frac{\mathrm{H_2/s}}{\mathrm{cm^2_{geo}}}\ \mathrm{per}\ \frac{\mathrm{mA}}{\mathrm{cm^2_{geo}}})\times|j|}{\text{No. of active sites}(\beta\text{-NiMoO}_4)\times A_{\mathrm{ECSA}}(\text{P-NiMoHZ})} \tag{7}$$

Supplementary Figures:

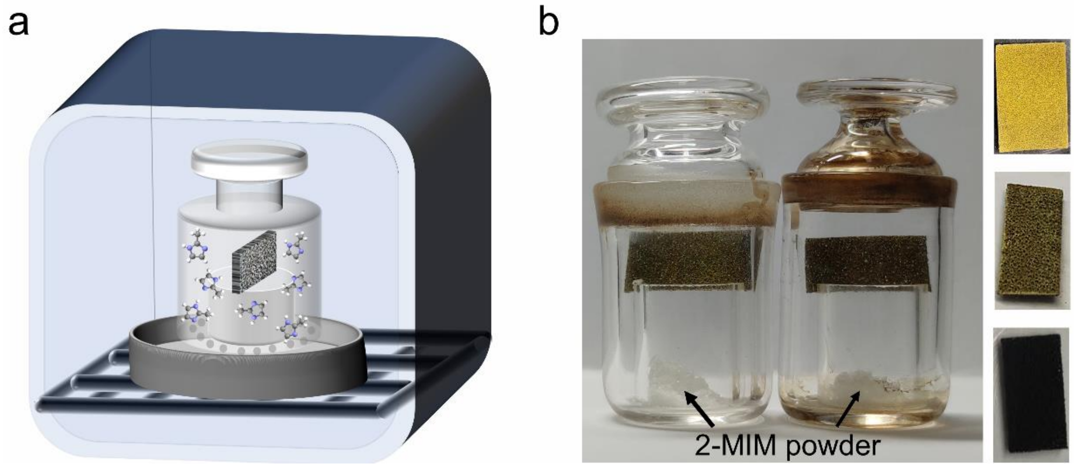

Supplementary Figure 1. (a) Scheme of synthesizing NiMoHZ by sublimation-vapor phase transformation device in an oven; (b) Photographs of the actual reaction device (left) and the prepared samples of $NiMoO_4 \cdot xH_2O$, NiMoHZ, and P-NiMoHZ from top to bottom, respectively (right).

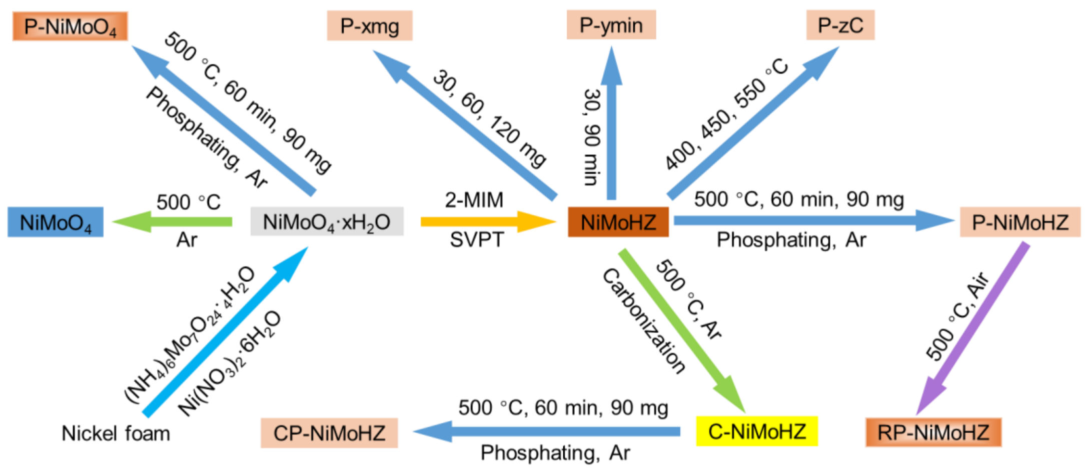

Supplementary Figure 2. The synthetic routes for the various samples in this work and the corresponding abbreviation names.

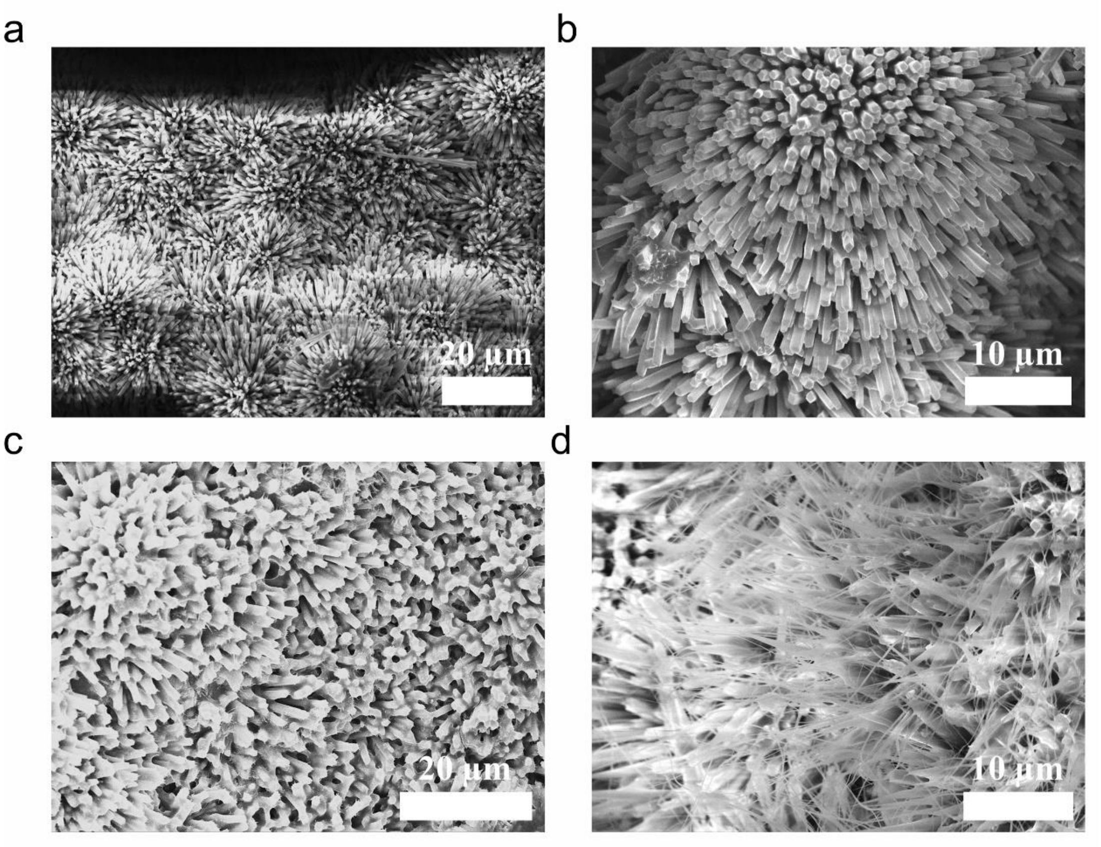

Supplementary Figure 3. SEM images of (a) precursor $NiMoO_4 \cdot xH_2O$ and transformed NiMoHZ with different reaction time: (b)1 h, (c) 2 h, (d) 3 h.

Basically, after 2 h SVPT reaction, a thin MOF coating forms on the surface of nanorod $NiMoO_4$ (Supplementary Fig.3c). However, when prolonging the reaction time to 3 h, the coating further grows into thread-like MOF crystals (Supplementary Fig.3d), which is consistent with the previously reported work.[3] In order to keep the fine nanorod structure, the 2 h sample was, therefore, chosen as our intermediate precursor for the following $NiMoO_4$ crystal phase conversion and named as NiMoHZ.

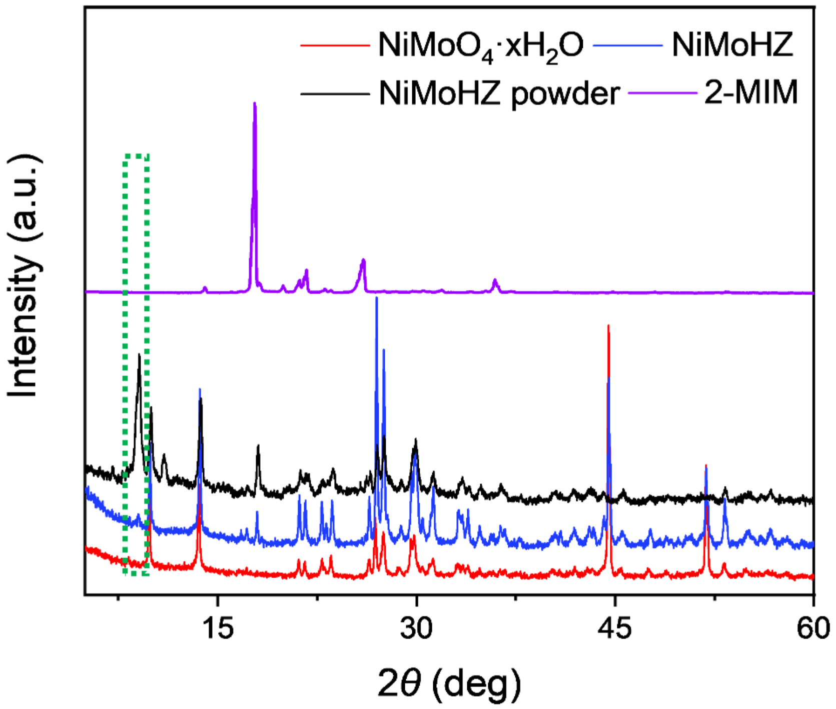

Supplementary Figure 4. XRD patterns of $NiMoO_4 \cdot xH_2O$, NiMoHZ, NiMoHZ powder, and 2-MIM powder. The peak in green marked area belongs to HZIF($Ni_4(im)_6MoO_4$).

According to the XRD results of SEM and XRD in Supplementary Fig. 3 and 4, the structure of NiMoHZ was determined as hydrate $NiMoO_4$ (inner nanorod) and hybrid zeolitic imidazolate framework (HZIF, outer thin coating) composite.

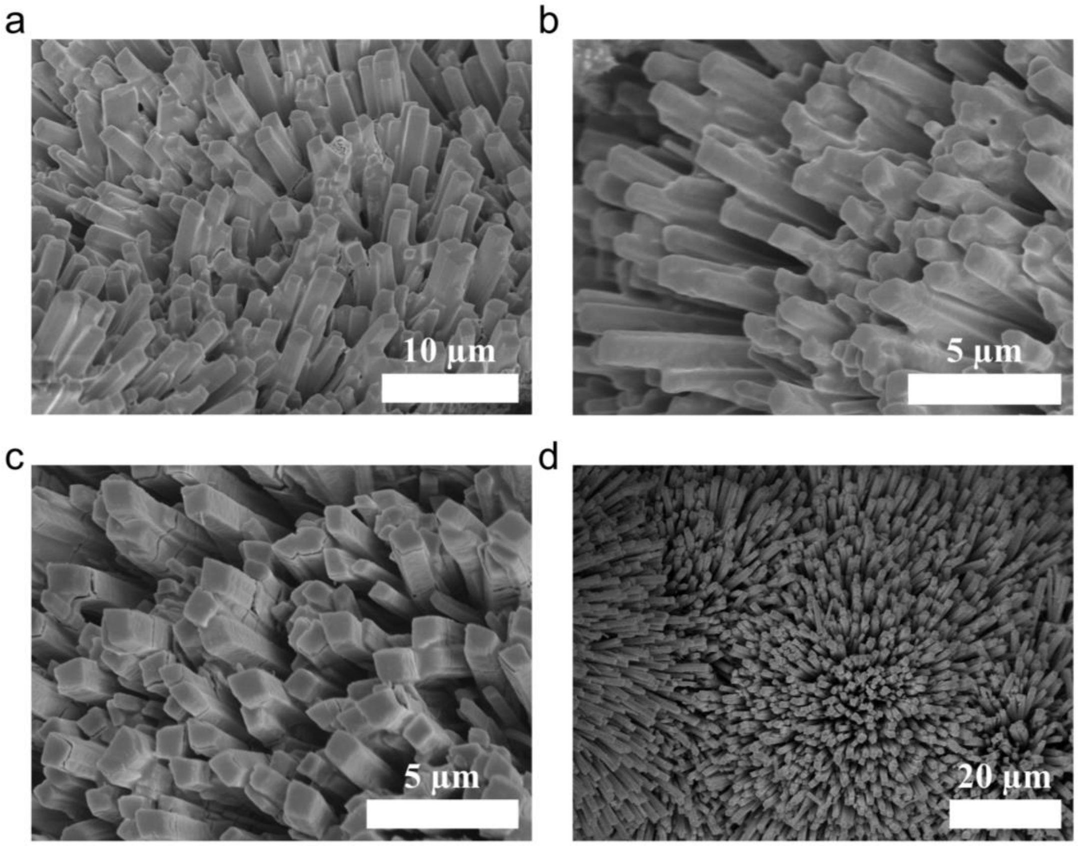

Supplementary Figure 5. SEM images of P-NiMoHZ control group samples obtained at different reaction temperatures: (a) 400 ºC, (b) 450 ºC, (c) 500 ºC; (d) SEM image of $NiMoO_4$.

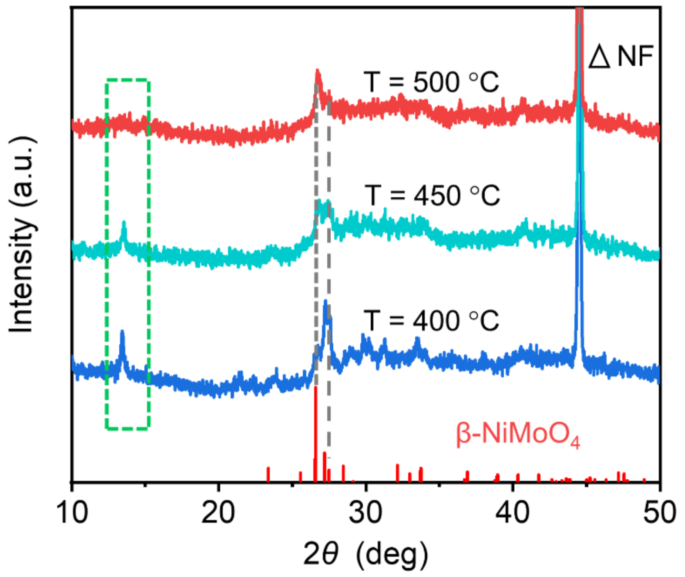

Supplementary Figure 6. XRD patterns of dehydrate $NiMoO_4$ and P-NiMoHZ samples obtained at different reaction temperature.

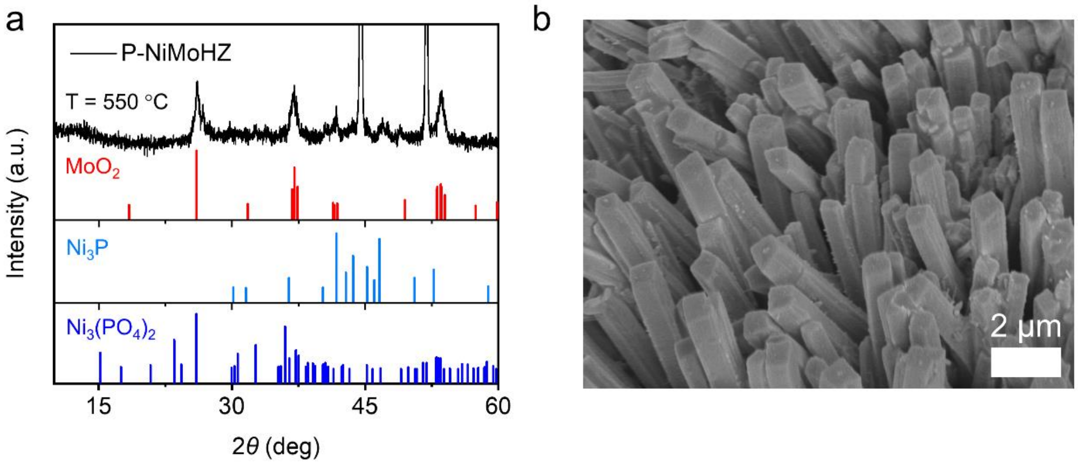

Supplementary Figure 7. (a) XRD pattern of P-NiMoHZ obtained at 550 ºC; (b) Corresponding SEM image.

The XRD pattern obtained at 550 ºC shows that excessive temperature would greatly increase the phosphating degree, making the precursor completely be converted into the composite of Ni_3P, $Ni_3(PO_4)_2$, and MoO_2 instead of β-$NiMoO_4$. The process follows by the below reaction equations:

$$8NiMoO_4 + 2PH_3 \rightarrow 8MoO_2 + Ni_3(PO_4)_2 + 5NiO + 3H_2O \quad (8)$$

$$57NiO + 22PH_3 \rightarrow 16Ni_3P + 3Ni_3(PO_4)_2 + 33H_2O \quad (9)$$

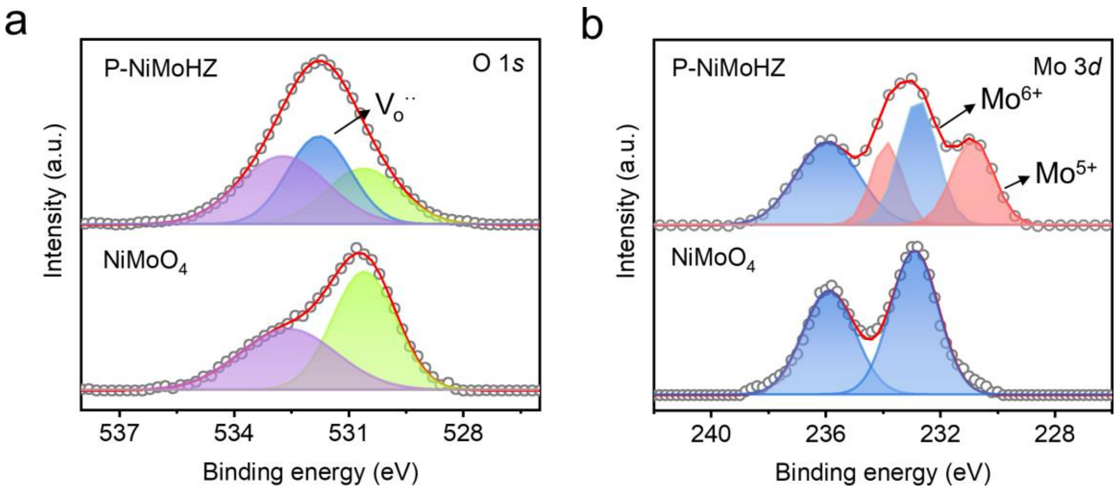

Supplementary Figure 8. XPS and experimental investigation into the stabilization mechanism of metastable phase P-NiMoHZ. XPS spectra of (a) O 1*s*, (b) Mo 3*d*.

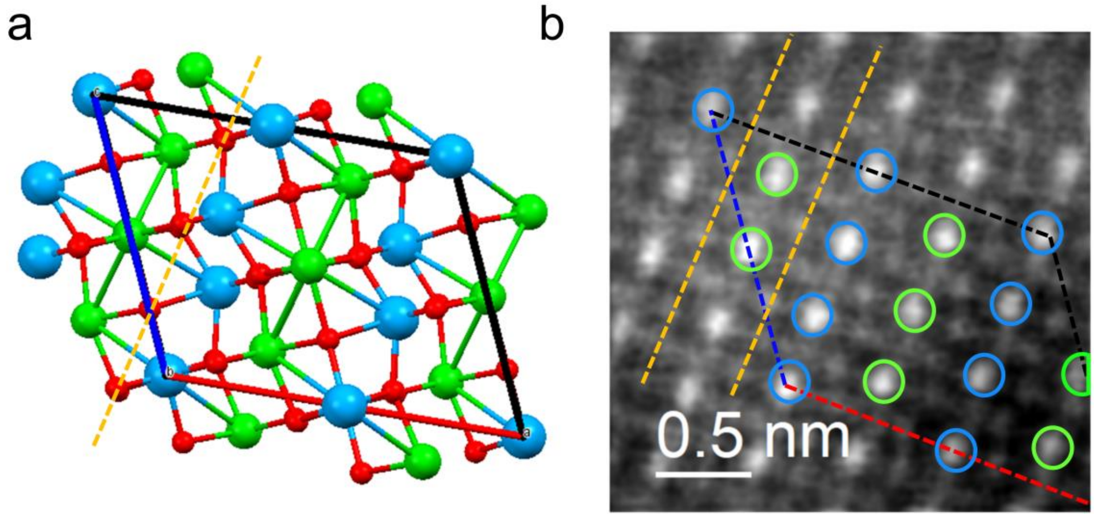

Supplementary Figure 9. (a) (101) plane model of α-$NiMoO_4$. The blue, green, and red balls represent Mo, Ni, and O atoms, respectively, while black, blue, and red lines represent cell axes. (b) High-resolution HAADF-STEM image of $NiMoO_4$ synthesized by dehydration of $NiMoO_4 \cdot xH_2O$. The bright dots represent metal atoms.

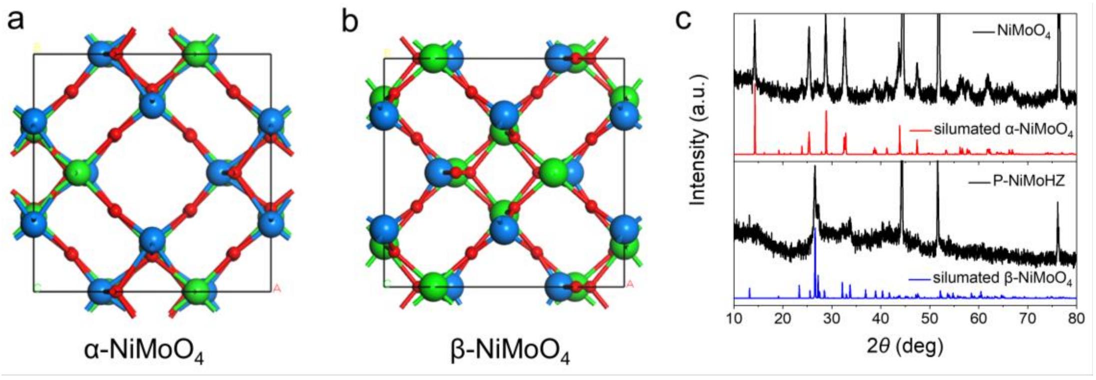

Supplementary Figure 10. Unit cell schematics of (a) α-$NiMoO_4$. (b) β-$NiMoO_4$, the blue, green, and red balls represent Mo, Ni, and O atoms, respectively. (c) Comparisons between simulated theoretical characteristic peaks and XRD patterns of $NiMoO_4$ and P-NiMoHZ.

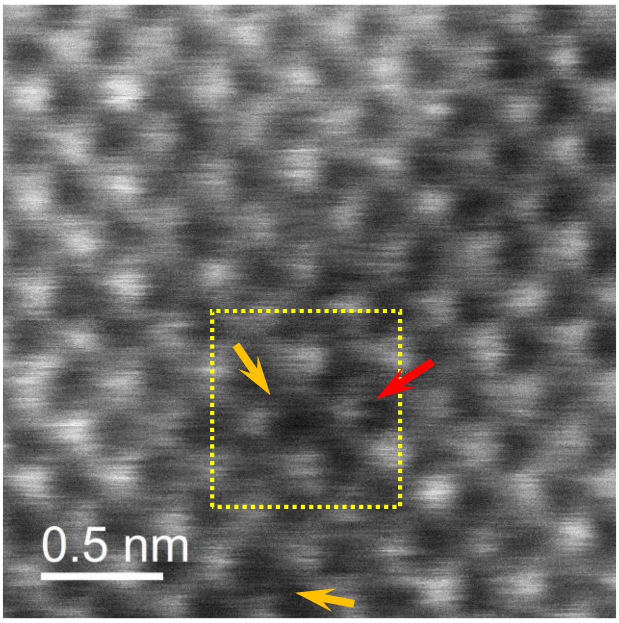

Supplementary Figure 11. Amplified HAADF-STEM image of P-NiMoHZ. V_o are marked by orange arrows, while P substitution by the red arrow.

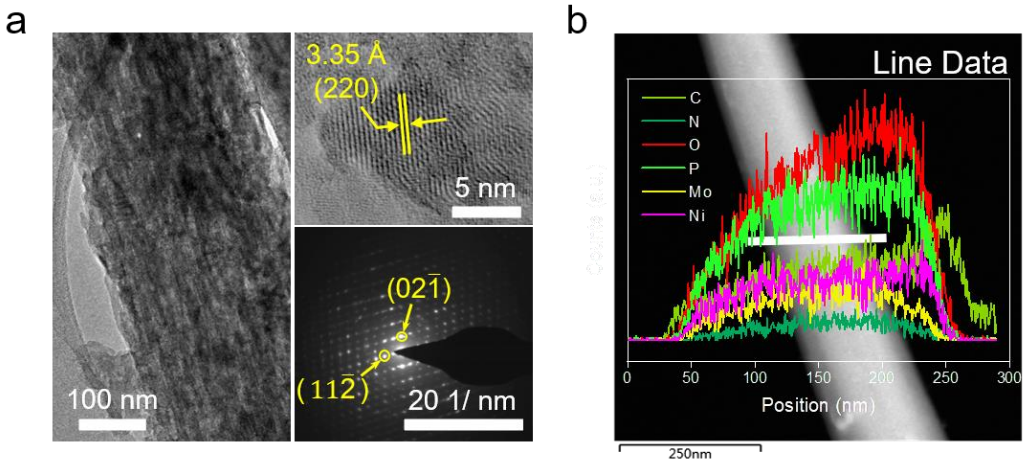

Supplementary Figure 12. (a) TEM, HR-TEM and SAED image of NiMoHZ; (b) Cross-sectional elemental distributions by line scans along the white line.

HR-TEM image shows the lattice fringe spacing of 3.35 Å, which is assigned to the (220) facet of β-$NiMoO_4$. The SAED image shows a single crystal diffraction pattern. The two marked bright spots are assigned to (11$\bar{2}$) and (02$\bar{1}$) plane of β-$NiMoO_4$.

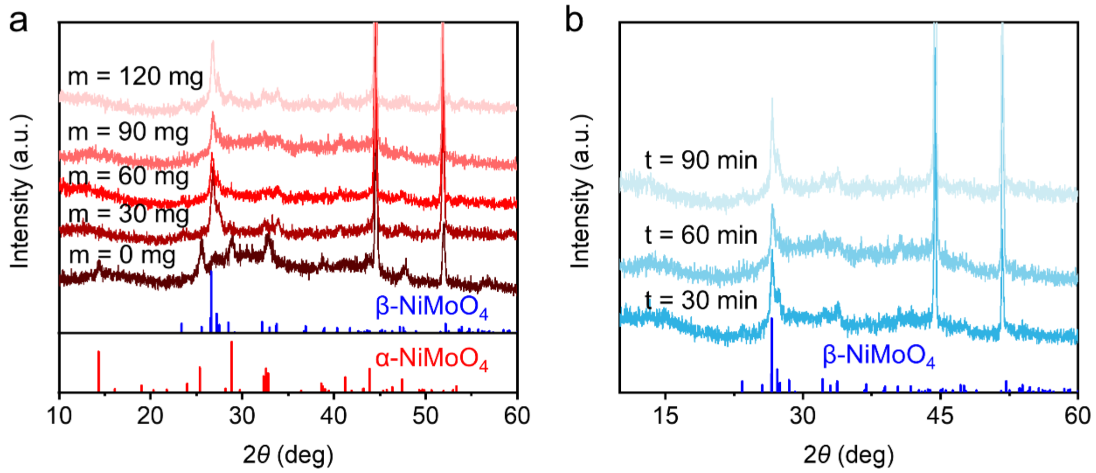

Supplementary Figure 13. XRD patterns of P-NiMoHZ control groups prepared under certain conditions: (a) different amount of $NaH_2PO_2 \cdot H_2O$. (b) different reaction time.

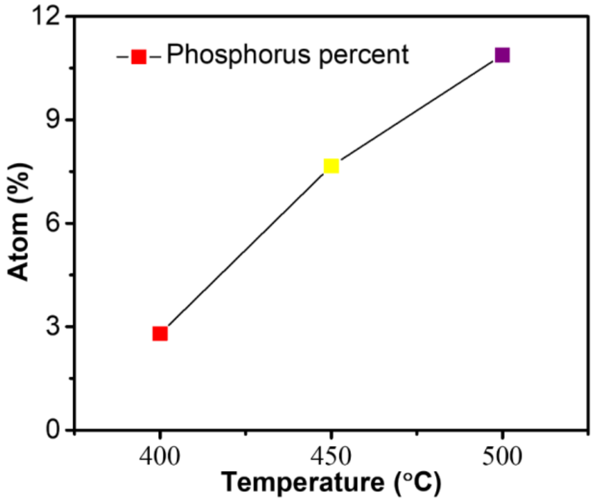

Supplementary Figure 14. Phosphorus atomic percent of P-NiMoHZ control group samples. The phosphorus atomic percent was summarized according to the results of EDX spectrum in Supplemental Table 1.

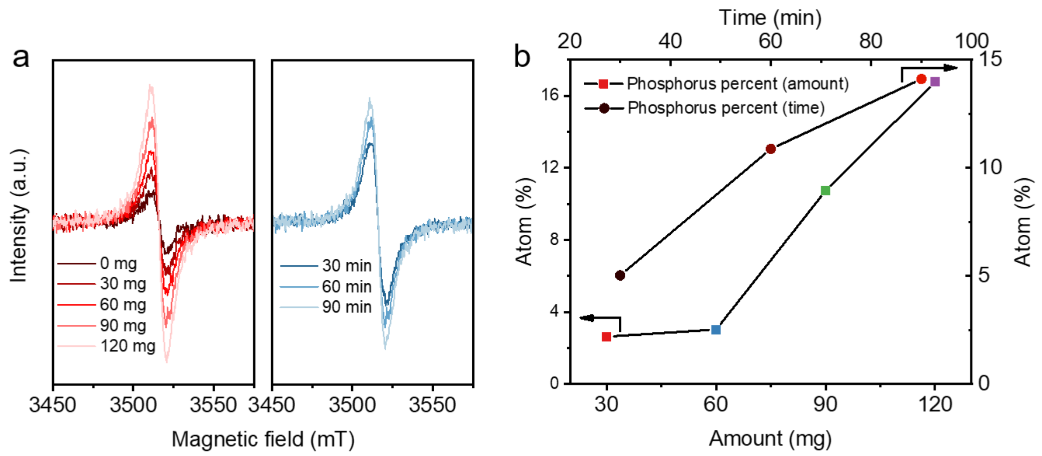

Supplementary Figure 15. (a) EPR spectra of P-NiMoHZ control group samples with different amount of $NaH_2PO_2 \cdot H_2O$ (left) and different reaction time (right). (b) Phosphorus atomic percent of these control groups.

The phosphorus atomic percent was summarized according to the results of EDX spectra in Supplemental Table 1. EPR spectra show a positive correlation relationship between the V_o and the P atomic content in the control group samples. Notably, C-NiMoHZ (without feeding of phosphate, m = 0 mg) has some oxygen vacancies although the amount is not as high as other control group samples.

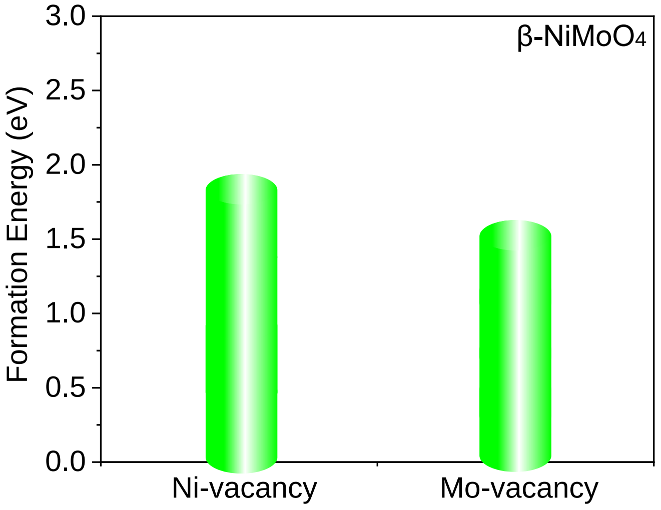

Supplementary Figure 16. The formation energies of proposed configurations of Ni and Mo vacancy in β-$NiMoO_4$ system. The formation energies are calculated as $E_f = E$ (β-$NiMoO_4$-$\square_x$) + xE (TM) – E (β-$NiMoO_4$) (TM= Ni or Mo, □ = Ni or Mo vacancy, x is the number of TM vacancy per unit cell).

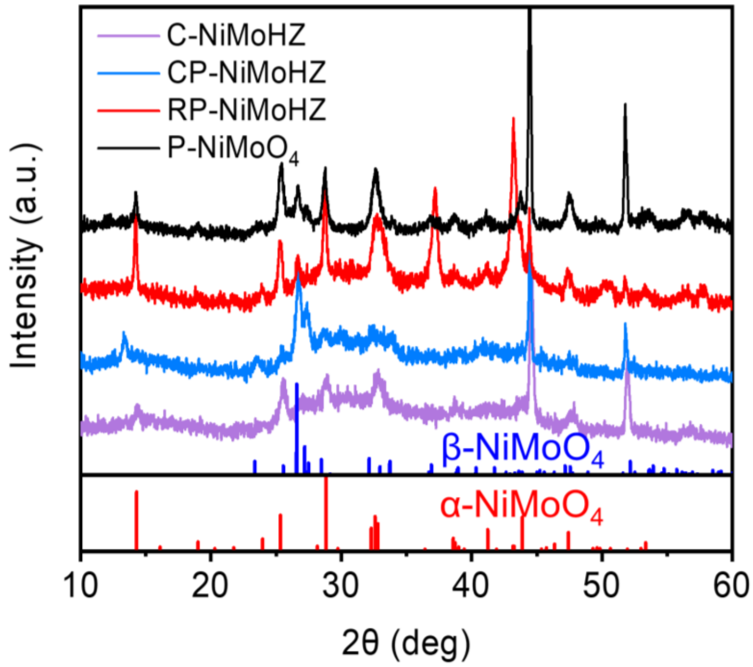

Supplementary Figure 17. The XRD patterns of C-NiMoHZ, CP-NiMoHZ (Phosphating C-NiMoHZ), RP-NiMoHZ (calcining P-NiMoHZ in air atmosphere) and P-$NiMoO_4$ (Phosphating $NiMoO_4$).

C-NiMoHZ is in the alpha phase with a certain amount of oxygen vacancies. After phosphating, it was converted into β-$NiMoO_4$ (CP-NiMoHZ). Furthermore, direct phosphating of $NiMoO_4$ could also obtain the β-$NiMoO_4$ but not pure β phase. β-$NiMoO_4$ would be partially converted back to α-$NiMoO_4$ if it is calcined in the air atmosphere (RP-NiMoHZ), which means that part of the oxygen vacancies would be compensated during the oxidative treatment. This result highlights the subordinate function of V_o in phase transformation mechanism.

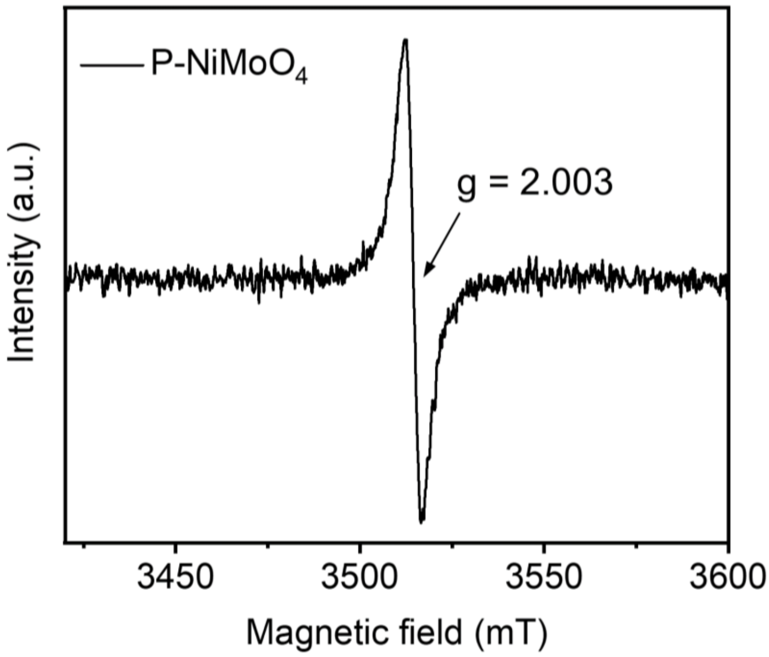

Supplementary Figure 18. EPR spectrum of P-$NiMoO_4$.

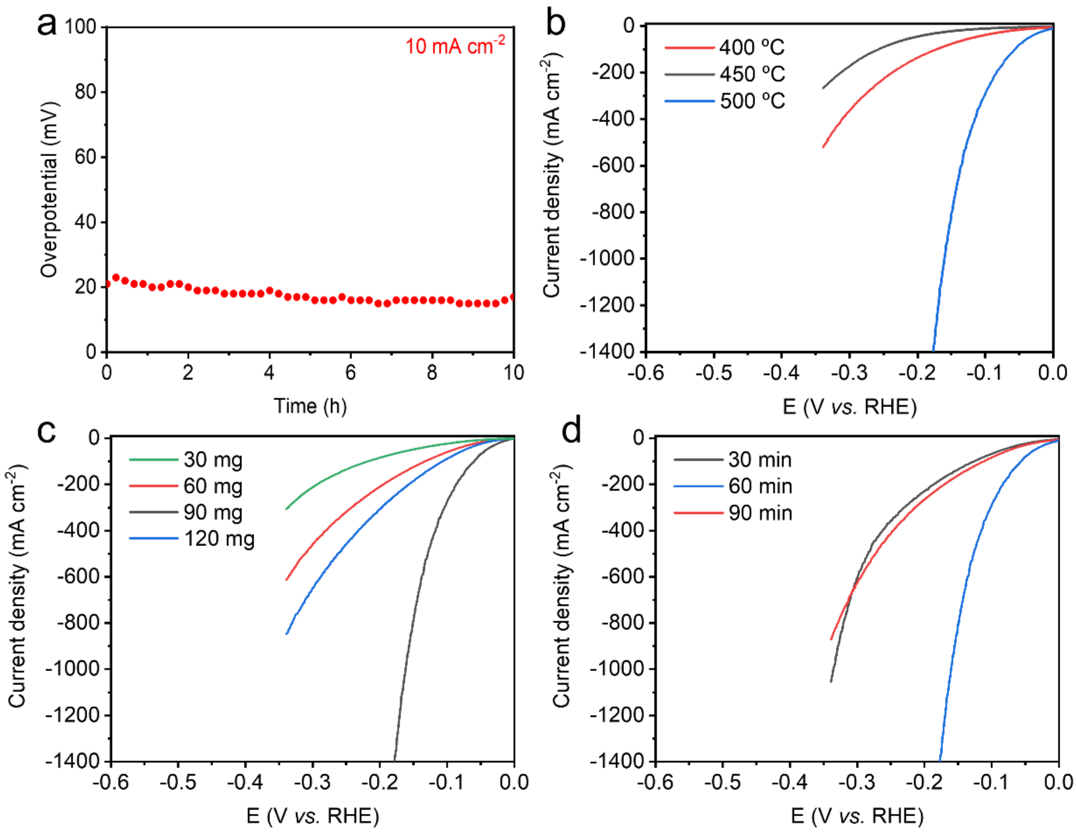

Supplementary Figure 19. (a) Chronopotentiometry curve of P-NiMoHZ for 10 h at 10 mA·cm^{-2}. HER polarization curves of P-NiMoHZ control group samples with (b) different temperature, (c) different amount of $NaH_2PO_2 \cdot H_2O$, and (d) different reaction time. The curves of 500 ºC, 90 mg, and 60 min were the same, which all belong to the optimal P-NiMoHZ sample.

P atomic percent and V_o amount order: P-30 mg<P-60 mg<P-90 mg<P-120 mg; P-30 min<P-60 min<P-90 min. (from the results in Supplementary Fig. 15)

HER activity order: P-30 mg<P-60 mg<P-120 mg<P-90 mg; P-30 min<P-90 min<P-60 min.

Based on the theoretical calculation results (Fig. 2b and Fig. 4), the active sites are the Ni atoms for H_2O dissociation and O1 sites connected with P and Ni for hydrogen desorption. Similar to previously reported works,[4-6] V_o is considered to adjust the electronic structure of β-$NiMoO_4$ and increase its conductivity. Therefore, when increasing the phosphating degree, the V_o amount would simultaneously increase, thus

both contributing to the great enhancement of the active site number and conductivity, while the crystal structure remains unchanged. As a result, the HER performance would accordingly be improved.

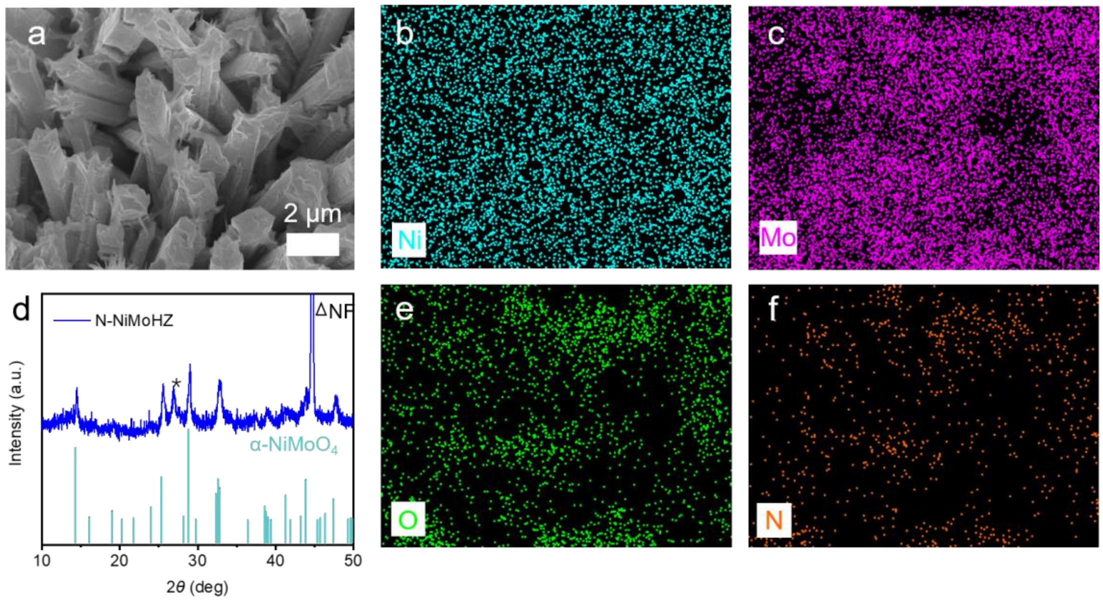

Supplementary Figure 20. (a) SEM image of N-NiMoHZ. Corresponding EDX elemental mapping of (b) Ni, (c) Mo, (e) O, and (f) N. (d) XRD pattern of N-NiMoHZ.

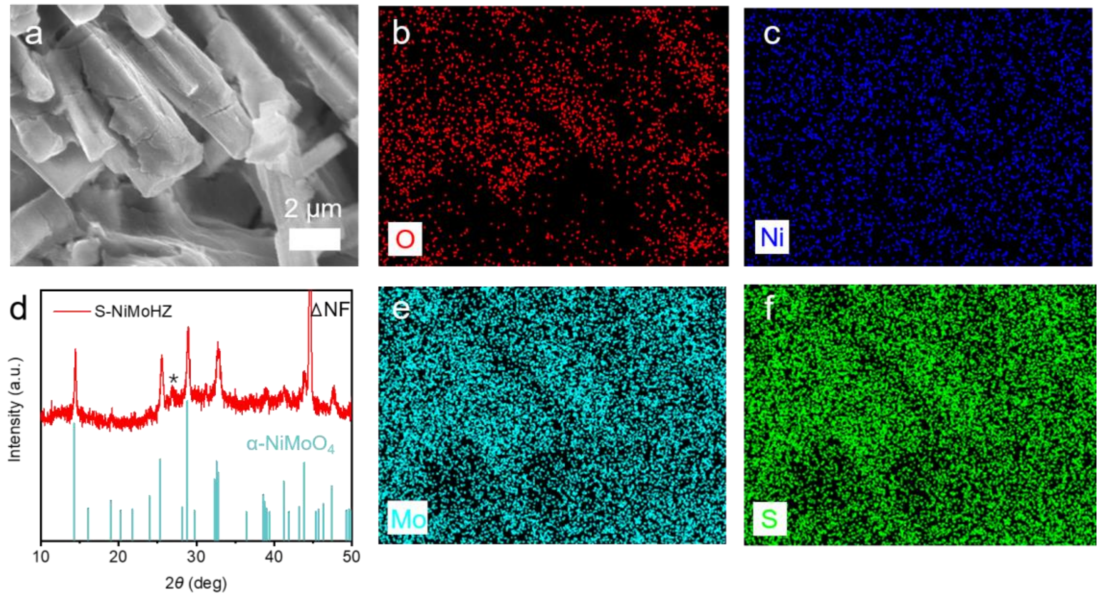

Supplementary Figure 21. (a) SEM image of S-NiMoHZ. Corresponding EDX elemental mapping of (b) O, (c) Ni, (e) Mo, and (f) S. (d) XRD pattern of S-NiMoHZ.

According to the XRD patterns in supplementary Fig. 20d and 21d, the as-prepared N-NiMoHZ and S-NiMoHZ are mostly in the form of a-$NiMoO_4$. The small peaks marked by black star are assigned to ammonium molybdenum oxide and nickel sulfide, respectively, which only account for a tiny part of the materials. Integrating the XRD and the energy dispersive X-ray (EDX) mapping results, it could be confirmed that N and S have successfully incorporated into the materials.

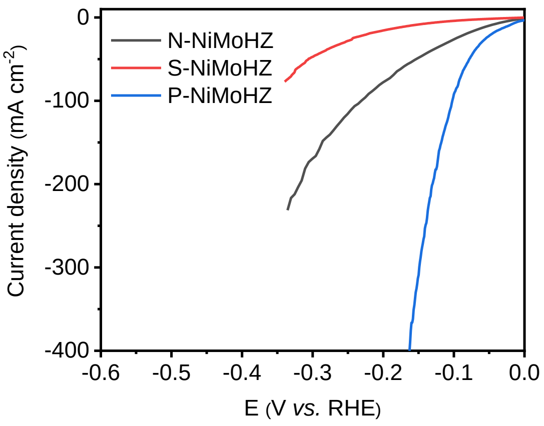

Supplementary Figure 22. Polarization curves of different non-metal-doping samples.

The HER performance of N-NiMoHZ and S-NiMoHZ is much worse than that of P-NiMoHZ because of the structure of P-β-$NiMoO_4$ in P-NiMoHZ. Generally, heteroatom-doping would improve the HER activity of the target electrocatalysts.[4,7-9] However, comparing these non-metal-doping samples, phosphate substituted β-$NiMoO_4$ plays a more important role in the enhancement of the HER performance, therefore displaying a much better HER performance.

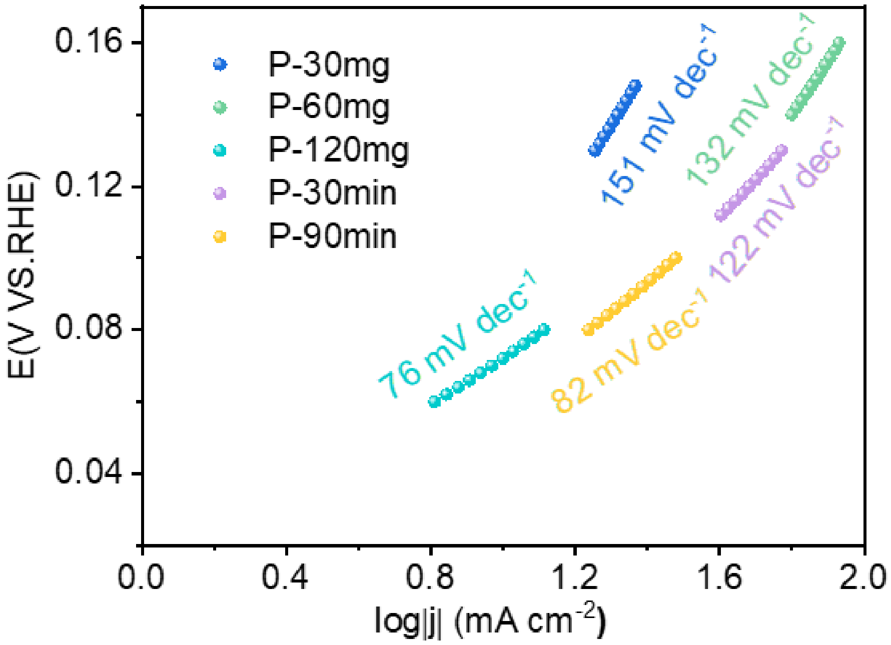

Supplementary Figure 23. The corresponding Tafel spots of different amounts of $NaH_2PO_2 \cdot H_2O$, different reaction times.

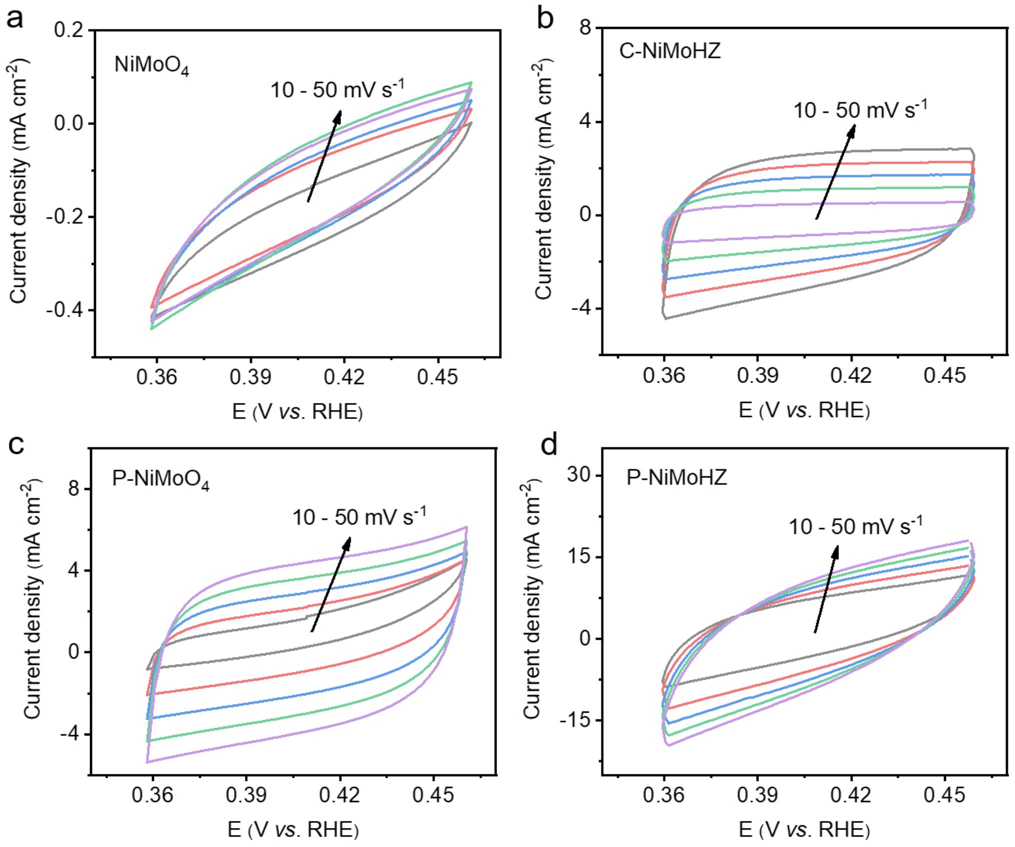

Supplementary Figure 24. CV curves of (a) $NiMoO_4$, (b) C-NiMoHZ, (c) P-$NiMoO_4$, and (d) P-NiMoHZ.

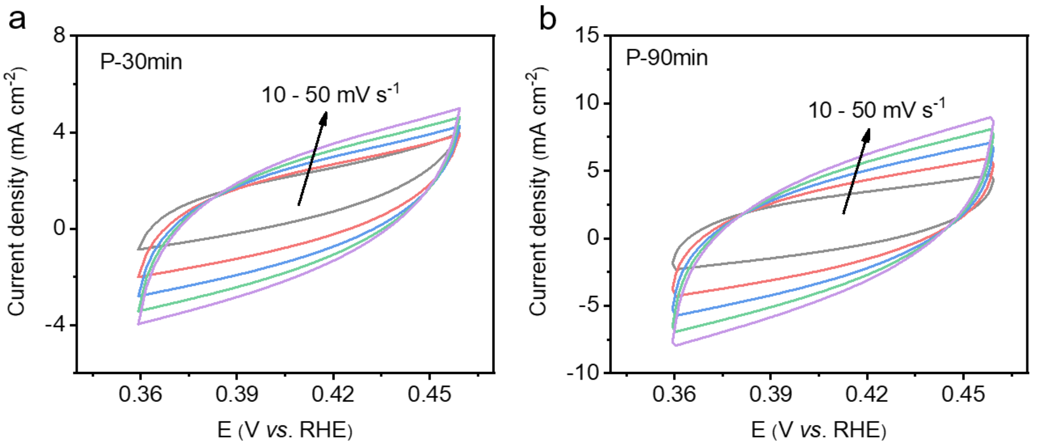

Supplementary Figure 25. CV curves of P-NiMoHZ control group samples: (a) P-30 min, (b) P-90 min.

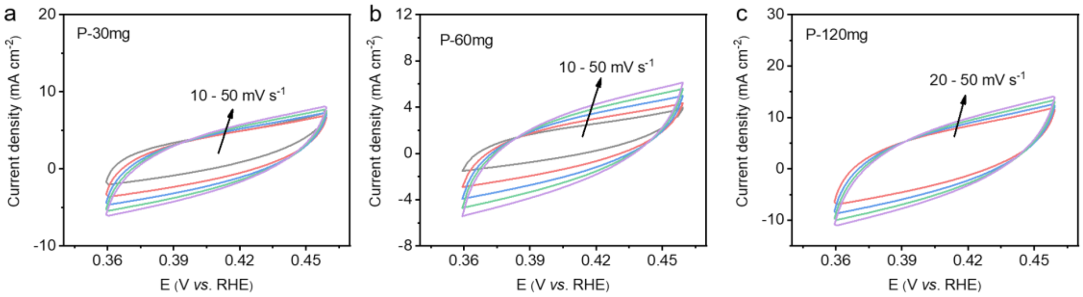

Supplementary Figure 26. CV curves of P-NiMoHZ control group samples: (a) P-30 mg, (b) P-60 mg and (c) P-120 mg.

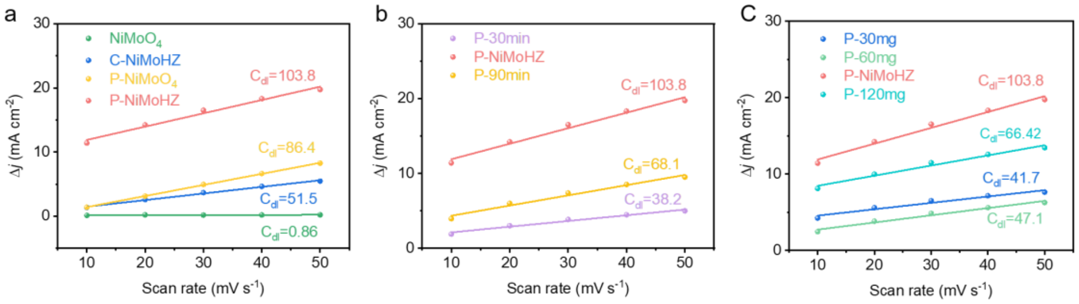

Supplementary Figure 27. C_{dl} values of (a) different crystal phase of $NiMoO_4$ and P-NiMoHZ control groups with different (b) reaction time, (c) amount of $NaH_2PO_2 \cdot H_2O$.

Since the potential window of -0.6 to -0.7 V vs. SCE (0.3594 to 0.4594 V vs. RHE, a range with no polarization) was selected to record CV curves, the middle point of 0.4094 V vs. RHE (-0.65 V vs. SCE) was chosen to calculate the difference between the anodic and cathodic current densities at different scan rate.[10,11]

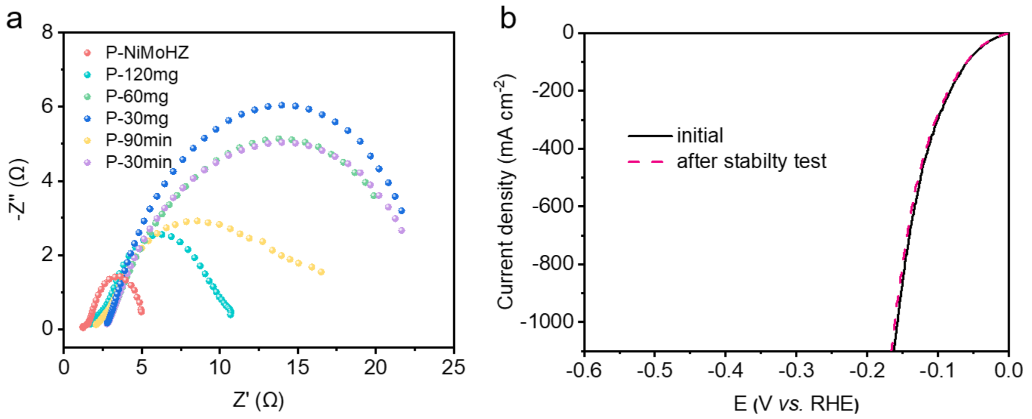

Supplementary Figure 28. (a) Nyquist plot of electrocatalysts P-NiMoHZ and control groups. (b) The polarization curves of P-NiMoHZ before and after stability test.

C_{dl} values (Supplementary Fig. 27b, c) and EIS results (Supplementary Fig. 28a) of P-NiMoHZ control group samples approve the relationship among active site number, conductivity, phosphating degree, and V_o amount until they reached the best HER performance. P-120 mg and P-90 min experienced a reverse change due to the excessive phosphate substitution and following local crystal distortion.

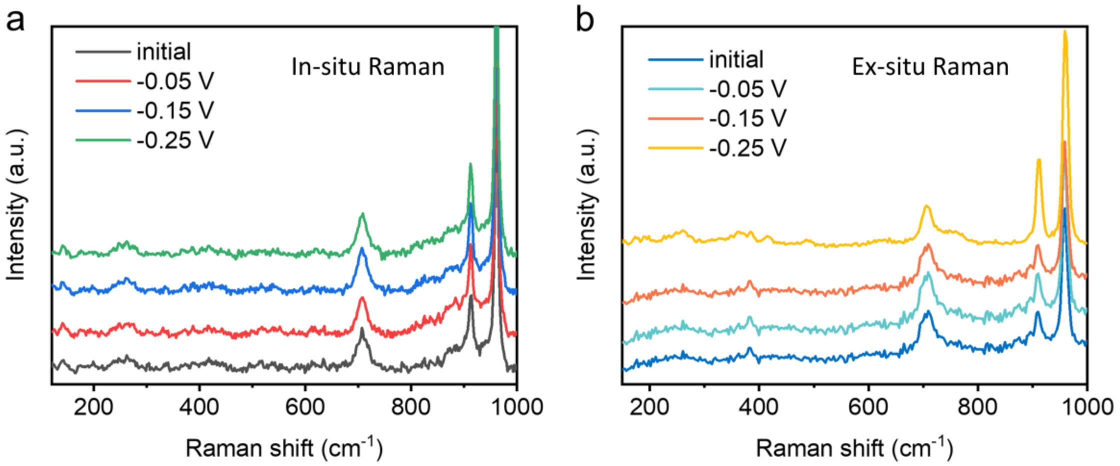

Supplementary Figure 29. (a) The potential-dependent in-situ Raman spectra of P-NiMoHZ during HER process. (b) The ex-situ Raman spectra of P-NiMoHZ after HER electrocatalysis at a certain potential.

In-situ Raman spectroscopy was applied to display the dynamic structure transformation of P-NiMoHZ at a series of potentials, ranging from 0 to -0.25 V vs. RHE. As shown in Supplementary Fig. 29a, the four patterns are almost the same. By comparing with the ex-situ Raman spectra of P-NiMoHZ (Supplementary Fig. 29b), in details, the peaks at 961.2 and 912.0 cm^{-1} are assigned to the symmetric and asymmetric stretching modes of the Mo=O bond, while the peak at 706.6 cm^{-1} belongs to the asymmetric stretching modes of Ni-Mo-O bonds.[12,13]

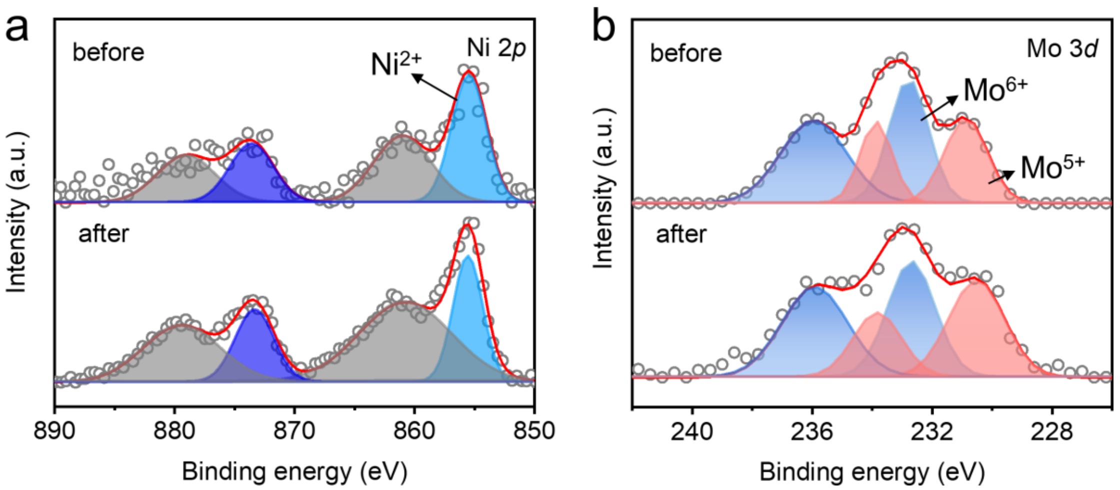

Supplementary Figure 30. (a) Ni 2*p* and (b) Mo 3*d* XPS spectra of the P-NiMoHZ before and after the stability test.

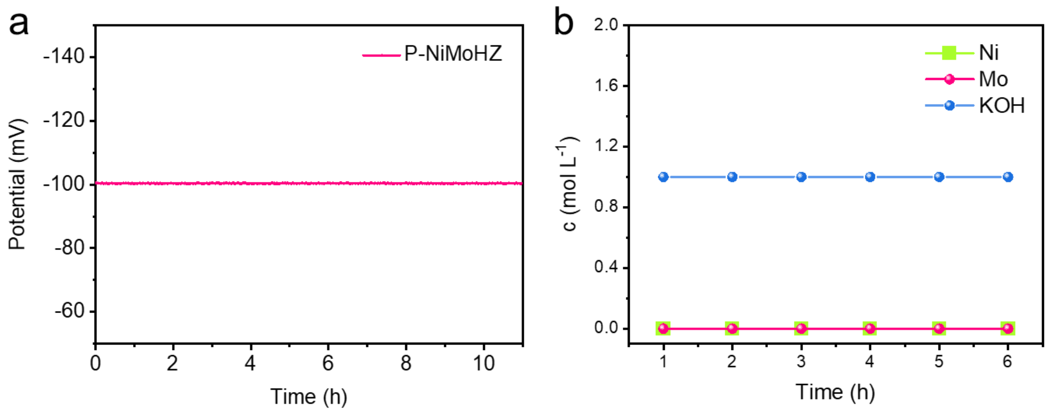

Supplementary Figure 31. (a) Long-term stability test of the P -NiMoHZ at 100 mA cm^{-2} and (b) time-dependent concentration the corresponding electrolyte.

As can be seen in Supplementary Fig. 31a, unlike the case of $NiMoO_4$ under OER condition, there is no surface reconstruction platform and step in this potential-time curve.[14] Through the analysis of the inductively coupled plasma-mass spectrometry (ICP-MS) (Supplementary Fig. 31b), it is clear that no material dissolution took place.

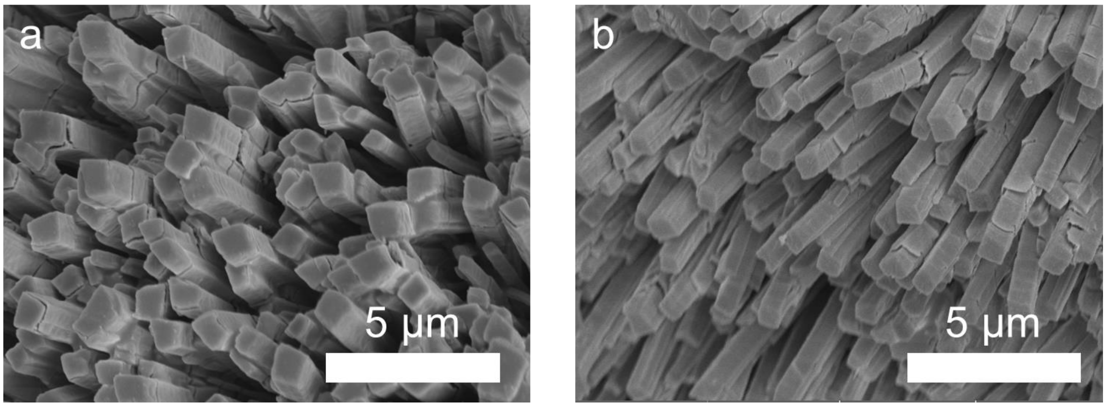

Supplementary Figure 32. SEM images of the P-NiMoHZ (a) before, and (b) after the stability test.

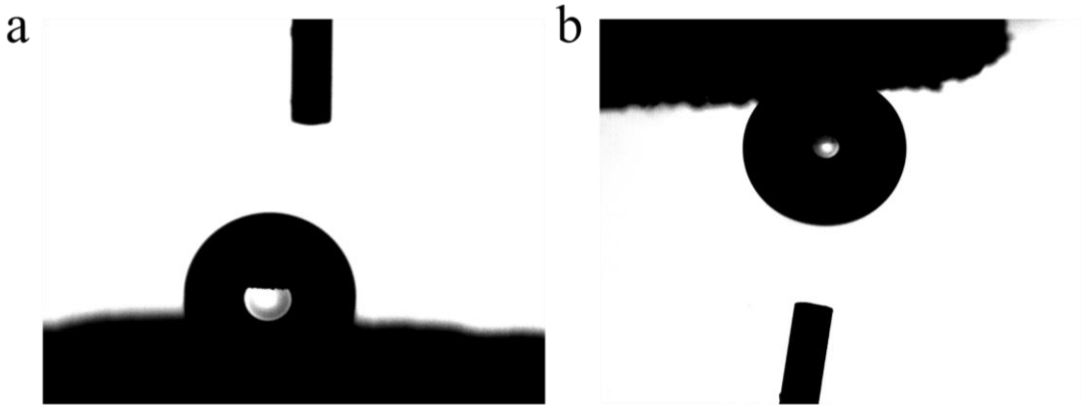

Supplementary Figure 33. Contact angle measurements for $NiMoO_4$ sample. (a) water contact angle, (b) H_2 contact angle. The water contact angle is 102º. The H_2 contact angle is 127º. These results indicate that $NiMoO_4$ is hydrophobic and more gas-philic than P-NiMoHZ.

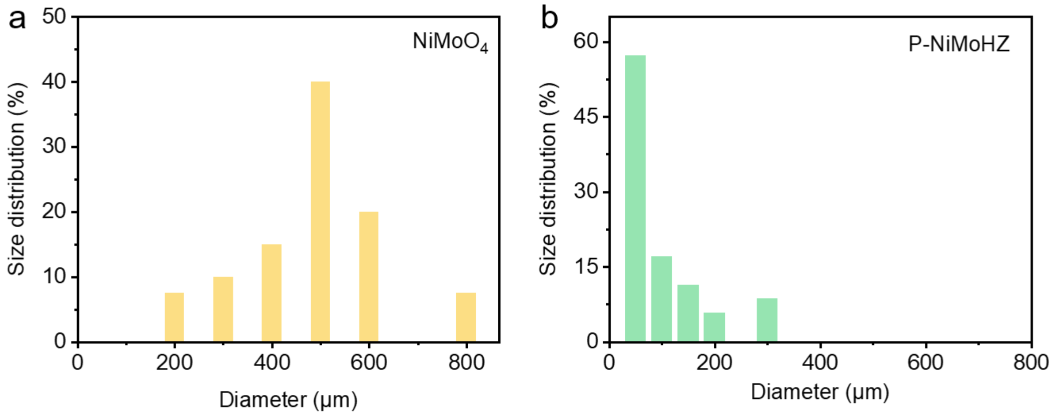

Supplementary Figure 34. Bubble size distributions on $NiMoO_4$ (a) and P-NiMoHZ (b) electrodes. In total forty bubbles were considered on each electrode.

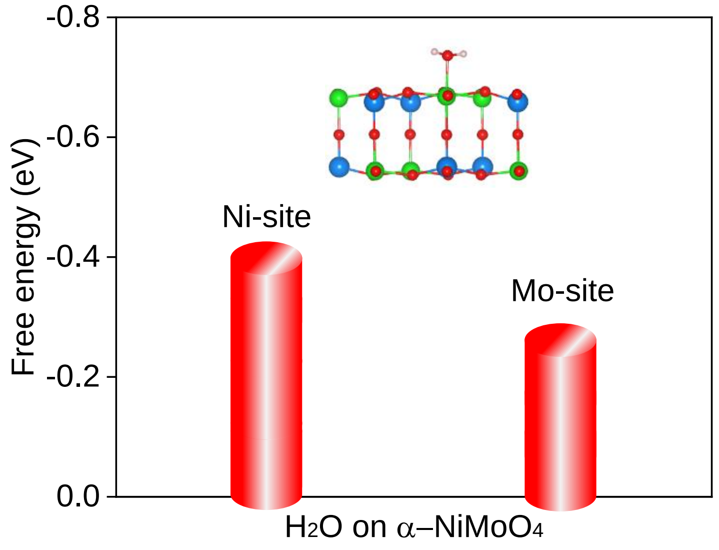

Supplementary Figure 35. H_2O adsorption free energies of possible sites in α-$NiMoO_4$. The Ni site of α-$NiMoO_4$ (110) exhibits relatively high adsorption energy.

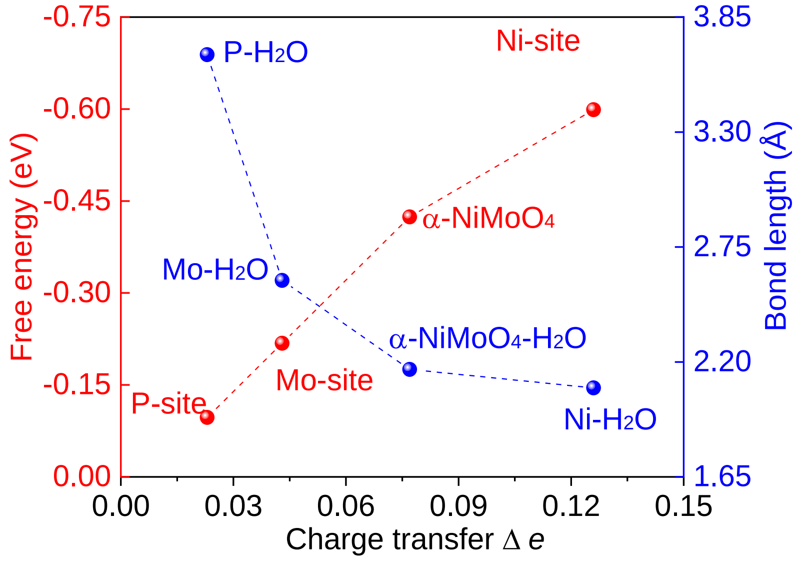

Supplementary Figure 36. Linear correlation between H_2O adsorption energy, P-β-$NiMoO_4$-H_2O bond length, α-$NiMoO_4$-H_2O bond length and the amount of charge transfer Δ *e* of various active sites in different phase of $NiMoO_4$ surface.

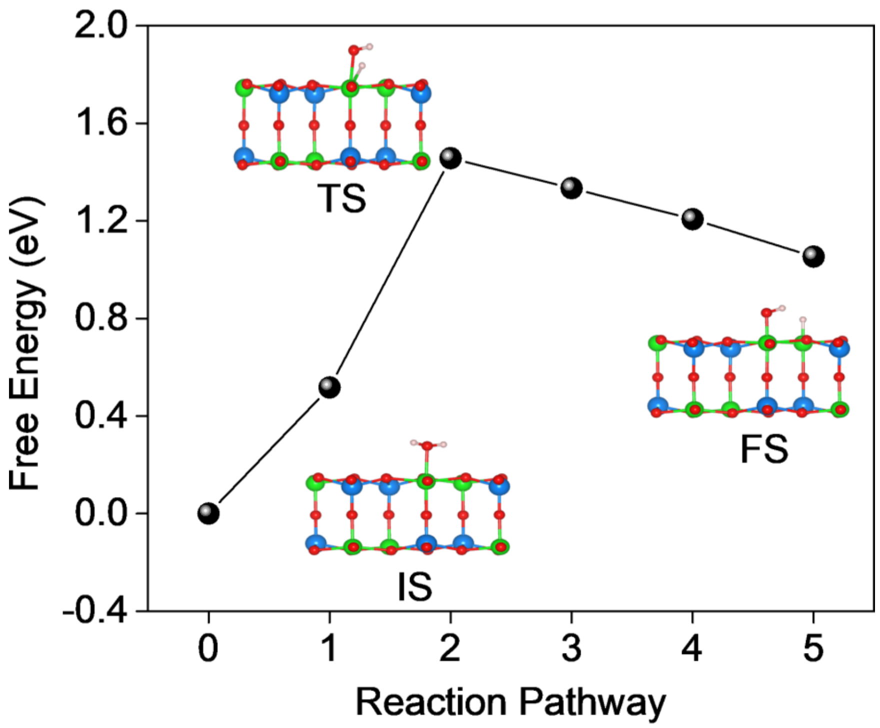

Supplementary Figure 37. Water dissociation barrier for reaction pathway of a-$NiMoO_4$ system. The insets are the structure of the corresponding IS (initial state), TS (transition state) and FS (final state). The colors of elements are: green for Ni, blue for Mo, red for O and white for H.

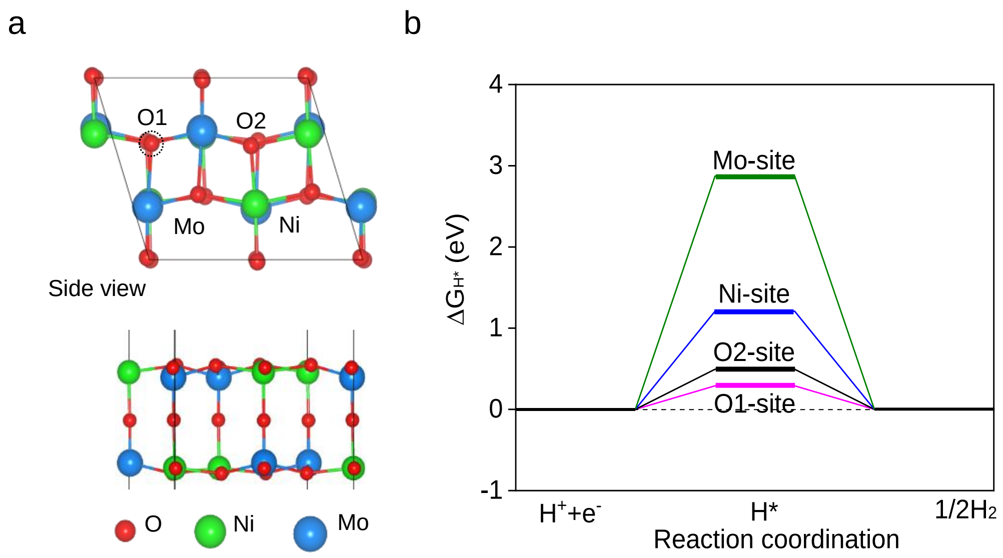

Supplementary Figure 38. Calculated hydrogen adsorption free energies of multiple active sites in α-$NiMoO_4$ (110). (a) Atomic configurations of simulated α-$NiMoO_4$ (110). (b) Hydrogen adsorption free energies (ΔG_{H*}) of possible sites. The O1 site of α-$NiMoO_4$ (110) exhibits relatively lower ΔG_{H*} for HER.

Supplementary Tables:

Supplementary Table 1. Elemental atomic percent contents of all control groups.

Controlled group samples	O (%)	Mo (%)	Ni (%)	P (%)	N (%)	Sum
P-30 mg	33.04	32.04	31.29	2.64	1.00	100
P-60 mg	46.17	24.97	23.89	3.03	1.94	100
P-NiMoHZ	61.79	11.60	13.94	10.73	1.95	100
P-120 mg	49.63	16.14	13.62	16.77	3.84	100
P-30 min	51.84	20.91	18.26	5.03	3.96	100
P-90 min	43.54	14.76	24.79	14.11	2.81	100
P-400 °C	62.06	16.92	18.17	2.8	0.06	100
P-450 °C	58.08	16.81	16.26	7.66	1.19	100

Supplementary Table 2. Hydrogen evolution rate of as-prepared electrocatalysts.

Samples	H_2 (mL h^{-1})	Mass loading (g cm^{-2})	H_2 (mL g^{-1} cm^{-2} h^{-1})
**P-NiMoHZ**	20.80	0.020	16640
P-$NiMoO_4$	13.20	0.022	9600
C-NiMoHZ	10.40	0.020	8320
$NiMoO_4$	8.40	0.019	7074

The calculated faradic efficiency (EF) is as high as 99%.

Supplementary Table 3. The comparison of TOF values at 100 mV overpotential of different electrocatalysts

Electrocatalyst	Electrolyte	TOF	Reference
**P-NiMoHZ**	1 M KOH	0.76	**This work**
$NiCo_2P_x$	1 M KOH	0.056	*Adv. Mater.*, **29**, 1605502 (2017)
Ni-MoS_2	1 M KOH	0.08	*Energy Environ. Sci.*, **9**, 2789 (2016)
Ni_5P_4	1 M KOH	0.06	*Energy Environ. Sci*. **8**, 1027−1034 (2015)
FeB_2	1 M KOH	0.165	*Adv. Energy Mater*. **7**, 1700513 (2017)
GDY/MoO_3	0.1 M KOH	0.22	*J. Am. Chem. Soc*. **143**, 8720−8730 (2021)
$NiCo_2P_x$	1 M KOH	0.056	*Adv. Mater.*, **29**, 1605502 (2017)
S-CoO NRs	1 M KOH	0.41	*Nat. Commun*. **8**, 1509 (2017)
CoN_x/C	1 M KOH	0.39	*Nat. Commun*. **6**, 7992 (2015)

Supplementary Table 4. The comparison of HER catalytic performance at large current density.

Electrocatalysts	Overpotential (mV)	Electrolyte	Reference
**P-NiMoHZ**	210 (-1000 mA cm^{-2})	1 M KOH	**This work**
Fe_3O_4/IF	240 (-1000 mA cm^{-2})	1 M KOH	*Adv. Mater.* **31**, e1905107 (2019)
MoS_2/Mo_2C	220 (-1000 mA cm^{-2})	1 M KOH	*Nat. Commun.* **10**, 269 **(**2019)
Ni_2P/NF	306 (-1000 mA cm^{-2})	1 M KOH	*J. Am. Chem. Soc.* **141**, 7537-7543 (2019)
2H $Nb_{1.35}S_2$	370 (-1000 mA cm^{-2})	1 M KOH	*Nat Mater.* **18**, 1309-1314 **(**2019)
Co_4N-CeO_2/GP	190 (-500 mA cm^{-2})	1 M KOH	*Adv. Funct. Mater.* **30**, 1910596 (2020)
MFN-MOFs/NF	234 (-500 mA cm^{-2})	1 M KOH	*Nano Energy.* **57**, 1-13 (2019)
NiFe-LDHs /MXene/NF	205 (-500 mA cm^{-2})	1 M KOH	*Nano Energy*. **63**, 103880 (2019)

Supplementary Table 5. The comparison of HER catalytic performance in 1 M KOH electrolyte at small current density.

Electrocatalysts	Overpotential (at 10 mA cm^{-2})	Tafel slope (mV dec^{-1})	Reference
**P-NiMoHZ**	23 mV	44	**This work**
PW-Co_3N	41 mV	40	*Nat. Commun.* **11**, 1853 (2020)
$NiCo_2P_x$	58 mV	34.3	*Adv. Mater.* **29**, 1605502 (2017)
$NiMo_3S_4$	257 mV	98	*Angew. Chem. Int. Ed.* **55**, 15240, (2016)
Ni-C-N	30.8 mV	40	*J. Am. Chem. Soc.* **138**, 14546 (2016)
CoMoP@C	81 mV	55.5	*Energy Environ. Sci.* **10**, 788 (2017)
$Ni_{0.33}Co_{0.67}Se_2$	106 mV	60	*Adv. Energy Mater.* **7**, 1602089 (2017)
$NiRu_{0.13}$-BDC	34 mV	32	*Nat. Commun.* **12**, 1369 (2021)
CP@Ni-P	117 mV	85.4	*Adv. Funct. Mater.* **26**, 4067 (2016)
Ni_3S_2@NPC	60.8 mV	67.5	*Nano Energy*, **36**, 85 (2017)
MoC_x	151 mV	59	*Nat. Commun.* **6**, 6512 (2015)
Mo_2C	270 mV	78	*J. Am. Chem. Soc.* **137**, 7035 (2015)
$MoS_{2(1-x)}Se_{2x}/NiSe_2$	69 mV	42.1	*Nat. Commun.* **7**, 12765 (2016)
P-Fe_3O_4/IF	50 mV	41.9	*Adv. Mater.* **31**, 1905107 (2019)
Sr_2RuO_4	61 mV	51	*Nat. Commun.* **10**, 149 (2019)
Mo-Co_9S_8@C	113 mV	67.6	*Adv. Energy Mater.* **10**, 1903137 (2020)
N-$NiMoO_4/NiS_2$	57 mV	74.2	*Adv. Funct. Mater.* **29**, 1805298 (2019)
$Ni_xCo_{2-x}P$@NC	37 mV	53.9	*Adv. Funct. Mater.* **29**, 1906316 (2019)
$Ni/Ni(OH)_2$	39 mV	53	*Adv. Mater.* **32**, 1906915 (2020)
SANi-PtNWs	65 mV	60.3(0.1M)	*Nat. Catal.* **2**, 495-503 (2019)

$Gd_{0.5}$	210 mV	29	*Nat. Commun*. **10**, 3755 (2019)
S-CoO NR	73 mV	82	*Nat. Commun*. **8**, 1509 (2017)

Supplementary Table 6. The comparison of HER performance at 10 mA cm^{-2} and 1000 mA cm^{-2} for NiMo-based and CoMo-based electrocatalysts.

Electrocatalyst	Overpotential (mV) at 10 mA cm^{-2}	Overpotential (mV) at 1000 mA cm^{-2}	Reference
**P-NiMoHZ**	23	210	**This work**
Ni_3N-NiMoN	31	/	*Nano Energy* **44**, 353–363 (2018)
Ni-Mo/GC1h	95	/	*ACS Catal.* **10**, 12858−12866 (2020)
PS-MoNi@NF	30	/	*Adv. Energy Mater.* **11**, 203511 (2021)
Mo-NiO/Ni	50	/	*ACS Energy Lett.* **4**, 3002−3010 (2019)
MoS_2/FNS/FeNi	120	/	*Adv. Mater.* **30**, 1803151 (2018)
MoS_2/Ni_3S_2	110	/	*Angew. Chem. Int. Ed.* **55**, 6702–6707 (2016)
Ni_4Mo	56	/	*Angew. Chem. Int. Ed.* **60**, 5771 –5777 (2021)
N–$NiCo_2S_4$	41	/	*Nat. Commun.* **9**, 1425 (2018)
$NiMoO_x$/NiMoS	38	236	*Nat. Commun.* **11**, 5462 (2020)
CoMoP@C	81	/	*Energy Environ. Sci.* **10**, 788 (2017)
$NiMo_3S_4$	254	/	*Angew. Chem. Int. Ed.* **55**, 15240 (2016)
H–Fe–CoMoS	137	/	*Nano Energy* **75**, 104913 (2020)
$Co_2Mo_3O_8$	38	/	*Nano Energy* **87**, 106217 (2021)

Supplementary References

1. Lu X, Zhao C. Electrodeposition of hierarchically structured three-dimensional nickel-iron electrodes for efficient oxygen evolution at high current densities. *Nat. Commun.* **6**, 6616 (2015).

2. Kibsgaard J*, et al.* Designing an improved transition metal phosphide catalyst for hydrogen evolution using experimental and theoretical trends. *Energy. Environ. Sci.* **8**, 3022-3029 (2015).

3. Chen J*, et al.* Sublimation-vapor phase pseudomorphic transformation of template-directed MOFs for efficient oxygen evolution reaction. *Adv. Funct. Mater.* **29**, 1903875 (2019).

4. Jin J*, et al.* Atomic sulfur filling oxygen vacancies optimizes H absorption and boosts the hydrogen evolution reaction in alkaline media. *Angew. Chem. Int. Ed.* **60**, 14117-14123 (2021).

5. Ling T*, et al.* Well-dispersed nickel- and zinc-tailored electronic structure of a transition metal oxide for highly active alkaline hydrogen evolution reaction. *Adv. Mater.* **31**, 1807771 (2019).

6. Zheng T*, et al.* Conductive tungsten oxide nanosheets for highly efficient hydrogen evolution. *Nano. Lett.* **17**, 7968-7973 (2017).

7. Wang HF*, et al.* MOF-derived electrocatalysts for oxygen reduction, oxygen evolution and hydrogen evolution reactions. *Chem. Soc. Rev.* **49**, 1414-1448 (2020).

8. Lin L*, et al.* Engineered 2D transition metal dichalcogenides—a vision of viable

hydrogen evolution reaction catalysis. *Adv. Energy. Mater.* **10**, 1903870 (2020).

9. Jin H*, et al.* Heteroatom-doped transition metal electrocatalysts for hydrogen evolution reaction. *ACS Energy Lett.* **4**, 805-810 (2019).

10. Shi H*, et al.* Spontaneously separated intermetallic Co_3Mo from nanoporous copper as versatile electrocatalysts for highly efficient water splitting. *Nat. Commun.* **11**, 2940 (2020).

11. Zhao Y*, et al.* Non-metal single-iodine-atom electrocatalysts for the hydrogen evolution reaction. *Angew. Chem. Int. Ed.* **58**, 12252-12257 (2019).

12. Solomon G*, et al.* $NiMoO_4$@Co_3O_4 core–shell nanorods: In situ catalyst reconstruction toward high efficiency oxygen evolution reaction. *Adv. Energy. Mater.*, 2101324 (2021).

13. Abdel-Dayem HM. Dynamic phenomena during reduction of α-$NiMoO_4$ in different atmospheres: In-situ thermo-raman spectroscopy study. *Ind. Eng. Chem. Res.* **46**, 2466-2472 (2007).

14. Liu X*, et al.* Reconstruction-determined alkaline water electrolysis at industrial temperatures. *Adv. Mater.* **32**, e2001136 (2020).
